# Supplementary figures and images for: Glycolytic Plasticity of Metastatic Lung Cancer Captured by Noninvasive 18F-FDG PET/CT and Serum 1H-NMR Analysis: An Orthotopic Murine Model Study
Source: Metabolites. 2023 Jan 9;13(1):110. doi: 10.3390/metabo13010110 (PMC9866275; doi:10.3390/metabo13010110)

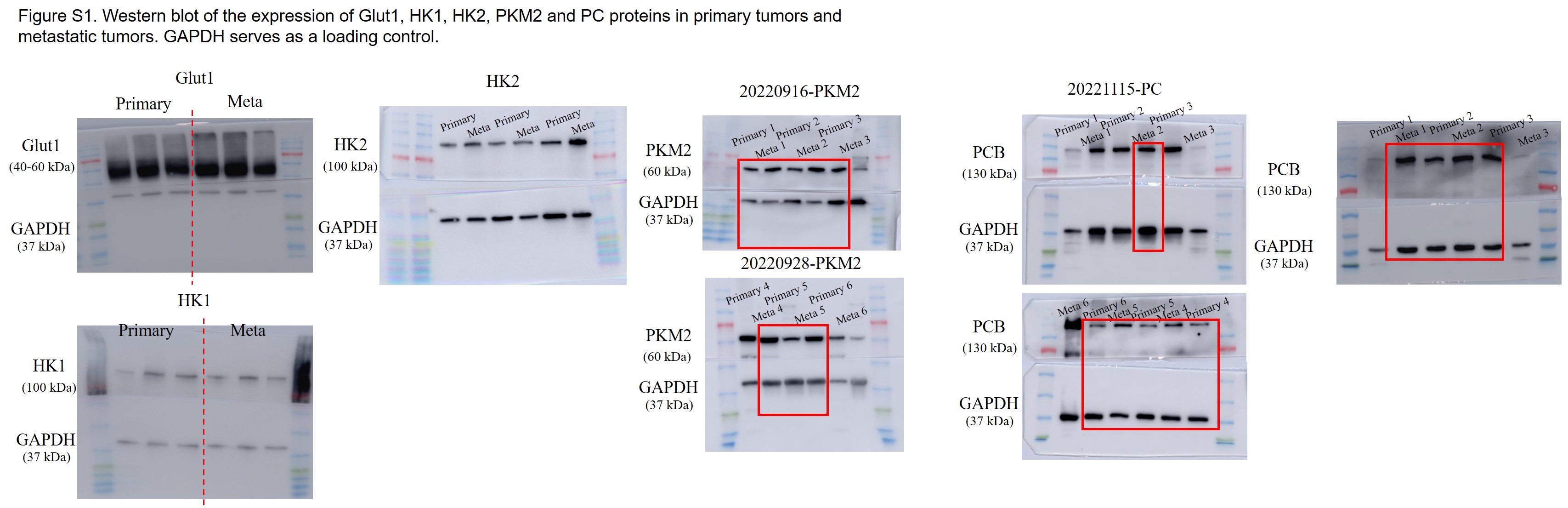

Supplement: Supplementary file 1 [file metabolites-13-00110-s001.zip › Figure S1.jpg]

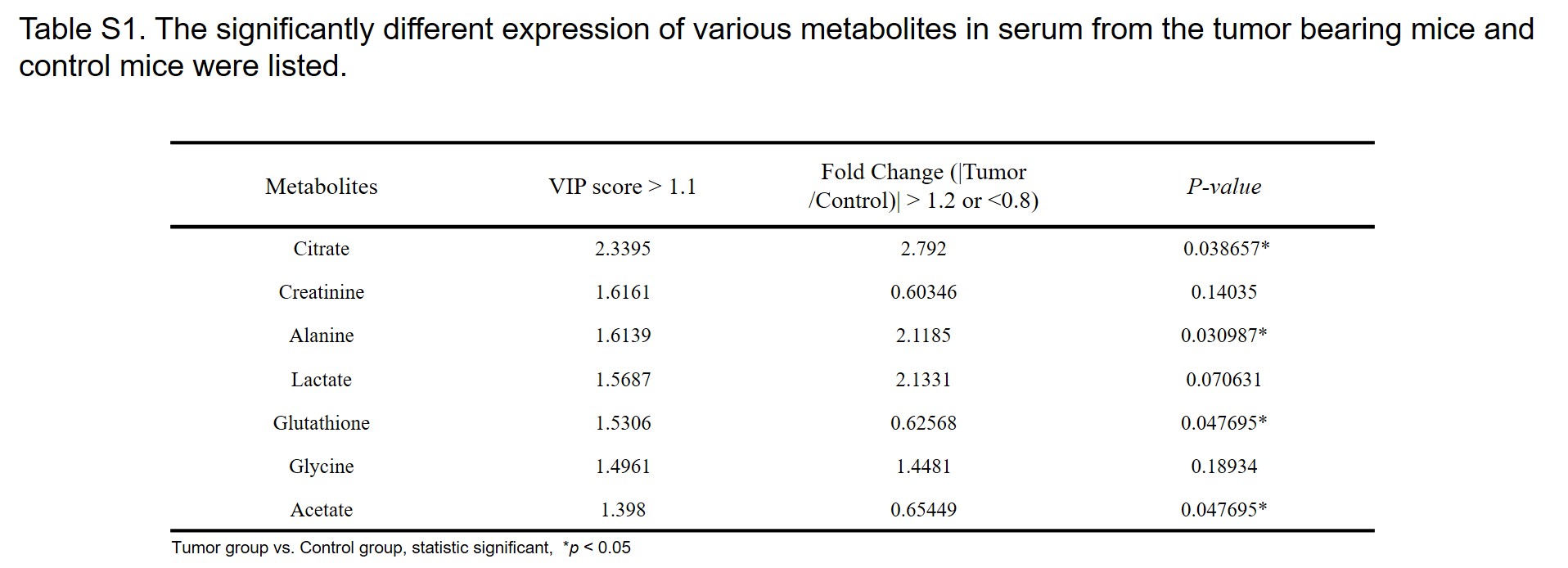

Supplement: Supplementary file 1 [file metabolites-13-00110-s001.zip › Table S1.jpg]
